# Proven prevention tools for addressing STI epidemics

**DOI:** 10.1186/s13584-018-0242-z

**Published:** 2018-08-06

**Authors:** Anatole S. Menon-Johansson

**Affiliations:** Burrell Street Sexual Health Clinic, Guy’s & St Thomas’ NHS Foundation Trust, 4-5 Burrell Street, London, SE1 0UN UK

## Abstract

The ongoing rise of sexually transmitted infections (STIs) poses a global public health challenge and the risk of acquiring one of these infections depends upon sexual practices, the number of sexual encounters and the location of that individual within the sexual network. Commercial sex workers (CSWs) have potentially a pivotal role in the transmission of STIs; however, a new study presented in this journal describes markers of risk but no increase in infections amongst men who pay for sex (MPS). This commentary highlights some of the growing evidence regarding STI prevention and the value of using these tools to protect CSWs, their clients and by extension the sexual partners of MPS.

The transmission of Sexually Transmitted Infections (STIs) depends upon the number of sexual partners, the number of concurrent partners and the coital frequency [1]. A recent special edition of the Lancet highlighted the importance of STIs, with an estimated 11 transmissions per second, and the challenges of STI control globally [2]. STI transmission between individuals depends upon their position in the sexual network: Commercial Sex Workers (CSWs) have the potential to spread infections to multiple Men who Pay for Sex (MPS) who in turn can further spread or ‘bridge’ these infections to subsequent sexual partners in lower risk sexual networks [3]. Since the majority of STI infections do not lead to noticeable symptoms, the unwitting transmission of infections to and from MPS has the potential to drive epidemics and studies have shown a higher rate of STIs in MPS [4, 5].

The paper by Rich et at in this journal [6] has looked at the number of men attending open access sexual health services in Israel over 7 years and found that 27% were MPS. This group of men reported more at-risk behaviours such as increased partner number, drug taking and a history of STI diagnoses than the men that did not pay for sex; however, in this analysis no additional STI diagnoses were made in MPS.

Across the World commercial sex work provides convenient sexual intimacy and Israel, like many nations, is grappling with the best way to legislate an industry that is associated with exploitation [7]. The CSWs can play an important public health role and the effective implementation of prevention strategies in this group has the potential to reduce the transmission of infections. Table 1 highlights a range of proven tools that reduce STI transmission during penetrative sex and CSWs should be supported to access and use all of them in their work.

**Table 1:** Shows the prevention strategies that have been developed to protect against sexually transmitted infections and their effectiveness

Sexually Transmitted Infection	Prevention strategies*	Effectiveness
Non-viral infections (Chlamydia trachomatis/Neisseria gonorrhoea/Trichomonas vaginalis/Mycoplasma genitalium/Ureaplasma urealyticum/Treponema pallidum)	•Condoms	•90% effective with perfect use [10]
	•Testing for STIs and treating when necessary	•100% effective unless there is poor adherence to treatment, reinfection or drug resistance
	•Partner notification	•Reducing onward transmission, reinfection and the cost to make an STI diagnosis [11]
Human papilloma virus (HPV)	•Condoms	•Up to 50% [12]
	•Circumcision	•50% [13]
	•HPV vaccination	•90% [14]
Hepatitis A virus (HAV)	•Condoms	•Not known
	•Hepatitis A vaccination	•93% [15]
Hepatitis B virus (HBV)	•Condoms	•Not known
	•Hepatitis B vaccination	• > 90% [16]
Herpes virus (HSV 1 & 2)	•Condoms	•24% [17]
	•Prophylactic treatment	•48% [17]
Human Immunodeficiency Virus (HIV)	•Condoms	•10% [18]
	•Circumcision	•57% [13]
	•Treatment as prevention	•93% [19]
	•Preexposure prophylaxis (PrEP)	• > 90% [20]

*Abstinence has not been included since it is a rare component of the CSW client relationship

Prevention of Human Papilloma, Hepatitis A & B Viruses with vaccination and Human Immunodeficiency Virus (HIV) with Pre-exposure prophylaxis ensures that the risk of acquisition and then onward transmission from a CSW would be negligible. Similarly, if the CSW is already HIV positive, then treatment as prevention would stop onward transmission of the virus to MPS. The role of condoms to prevent bacterial and parasitic infections is still key as a public health intervention and CSWs need to be supported to use them for vaginal, anal and oral sex.

The biggest burden of poor sexual health in the future will come from non-viral infections and drug resistance is a growing challenge within these organisms [2]. Regular STI testing of ‘at risk’ individuals is important to identify infections within a sexual network and this needs to be complemented with effective partner notification to limit onward spread of these infections.

Finally, a history of sex with CSWs was not shown to be associated with acquisition of an STI in one Israeli clinic over 7 years. It is likely that the use of condoms played a pivotal role in this outcome and the high prevalence of circumcision within the men in this study is likely to have contributed too [8]. It is important to build upon these results and focus on CSW access to the prevention methods cited in the table as well as address prejudice against CSWs and their clients [9].

## Conclusions

There are a number of proven prevention tools for STI epidemics. The challenge is to ensure that patient education, staff training, and health care policy initiatives focus on the full use of prevention tools to improve the publics’ health.
